# The Mitochondrial Genome of *Paramphistomum cervi* (Digenea), the First Representative for the Family Paramphistomidae

**DOI:** 10.1371/journal.pone.0071300

**Published:** 2013-08-22

**Authors:** Hong-Bin Yan, Xing-Ye Wang, Zhong-Zi Lou, Li Li, David Blair, Hong Yin, Jin-Zhong Cai, Xue-Ling Dai, Meng-Tong Lei, Xing-Quan Zhu, Xue-Peng Cai, Wan-Zhong Jia

**Affiliations:** 1 State Key Laboratory of Veterinary Etiological Biology, Key Laboratory of Veterinary Parasitology of Gansu Province, Key Laboratory of Veterinary Public Health of Agriculture Ministry, Lanzhou Veterinary Research Institute, Chinese Academy of Agricultural Sciences, Lanzhou, Gansu Province, PR China; 2 School of Marine and Tropical Biology, James Cook University, Queensland, Australia; 3 Laboratory of Plateau Veterinary Parasitology, Veterinary Research Institute, Qinghai Academy of Animal Science and Veterinary Medicine, Xining, Qinghai Province, PR China; 4 College of Veterinary Medicine, Northwest A&F University, Yangling, Shanxi Province, PR China; Washington State University, United States of America

## Abstract

We determined the complete mitochondrial DNA (mtDNA) sequence of a fluke, *Paramphistomum cervi* (Digenea: Paramphistomidae). This genome (14,014 bp) is slightly larger than that of *Clonorchis sinensis* (13,875 bp), but smaller than those of other digenean species. The mt genome of *P. cervi* contains 12 protein-coding genes, 22 transfer RNA genes, 2 ribosomal RNA genes and 2 non-coding regions (NCRs), a complement consistent with those of other digeneans. The arrangement of protein-coding and ribosomal RNA genes in the *P. cervi* mitochondrial genome is identical to that of other digeneans except for a group of *Schistosoma* species that exhibit a derived arrangement. The positions of some transfer RNA genes differ. Bayesian phylogenetic analyses, based on concatenated nucleotide sequences and amino-acid sequences of the 12 protein-coding genes, placed *P. cervi* within the Order Plagiorchiida, but relationships depicted within that order were not quite as expected from previous studies. The complete mtDNA sequence of *P. cervi* provides important genetic markers for diagnostics, ecological and evolutionary studies of digeneans.

## Introduction

Paramphistomosis, due to paramphistomes (Trematoda: Digenea: Paramphistomidae), has recently emerged as a major cause of productivity loss in ruminants. Adult worms often inhabit the rumen and reticulum of cattle, water buffaloes, sheep and goats. Their presence in these sites may elicit few apparent signs or symptoms. However, acute parasitic gastroenteritis causing high morbidity and mortality may occur as large numbers of immature paramphistomes migrate through the intestine towards the rumen and reticulum. Severity of disease is greatest in young animals [1]–[6]. As a consequence of frequent under-diagnosis, the significance of subclinical infection in many animals remains unclear and economic losses may exceed those caused by many other helminth parasites [5], [7]. Paramphistomosis is widespread [6]–[10], with different species predominating in different places. However, *Paramphistomum cervi* is perhaps the most widespread species, being reported from many parts of Eurasia [9], [11] and North America [12]. Conventional diagnosis of paramphistomosis is based on the history and clinical signs of the disease. Further confirmation can be obtained by collection of fecal samples from the host and examination for parasite eggs. However, this can lead to misinterpretation or misdiagnosis because the presence of adult paramphistomes (hence their eggs) is not necessarily a cause of disease [7], [13], [14]. Early diagnosis of paramphistomosis is essential for prompt treatment before irreparable damage to the rumen and bile ducts occurs [9]. Immunological diagnosis may be a dependable means for monitoring the infection, and be supplemented by the finding of eggs. In order to develop this method, whole worm extract of adult *P. cervi* has been subjected to immuno-blotting using sera from bovines infected with *P*. *cervi*. This method, however, has not been widely adopted [15].

Rapid development of molecular biology techniques, especially the polymerase chain reaction (PCR), may provide valuable supplementary tools for the differential identification of digenean infection to overcome limitations of current morphological-diagnostic methods. Due to their high nucleotide substitution rates, partial or complete mitochondrial (mt) genomes of parasitic flatworms have become very popular markers for detecting their presence in animals and for investigating their phylogenetic relationships at different levels [16]–[29].

The lack of knowledge of mt genomics for *P*. *cervi* is a major limitation for the development of molecular diagnostic techniques, for analyses of population and genetic variation within this species, and for phylogenetic studies of the Digenea in general.

In our present study, we determined the complete mt nucleotide sequence of *P*. *cervi*, which was collected from Qinghai Province, China. Phylogenetic analyses were performed using concatenated mt sequences of 12 protein-coding genes of digenean species available in GenBank to date. The new mt genome sequence may provide useful information on both genomics and the evolution of Paramphistomidae, because there are no complete (or nearly complete) mtDNA sequences available from any member of this family.

## Materials and Methods

### Ethics Statement

The yak from which *P*. *cervi* adults were collected was being processed at a local abattoir in Dari County, Qinghai Province, as part of the normal work of the abattoir.

### Parasite and DNA extraction

Adult *P*. *cervi* (Zeder, 1790) were collected from the rumen of a naturally infected yak in Dari County, Qinghai Province of China. The flukes were washed extensively in physiological saline and identified to species in the Key Laboratory of Veterinary Parasitology, Gansu Province based on morphological characters (collection accession number: 20110101).

Total genomic DNA was extracted from one parasite using a Qiagen Blood and Tissue Kit (Qiagen, Germany) according to the manufacturer's instructions and eluted into 100 μl H_2_O, followed by RNase treatment step. The treated DNA sample was stored at −20°C until use.

### Amplification, sequencing and assembling of mtDNA fragments

Amplification, sequencing and assembly of mtDNA fragments was performed according to methods previously described [23], [24]. Seven pairs of oligonucleotide primers were designed based on the conserved regions from published complete mtDNA sequences of *Fasciola hepatica* [27], [28], *Clonorchis sinensis* [30], [31], *Opisthorchis felineus* [30] and *Paragonimus westermani* (GenBank Accession No. AF219379) (Table 1). These sets of primers amplified overlapping fragments to facilitate eventual assembly using *Taq* polymerase – KOD FX Neo (TOYOBO, Japan). The cycling conditions used were 94°C for 5 min (initial denaturation); then 94°C for 1 min (denaturation), 50°C for 35 s (annealing), 72°C for 1–3 min (extension) for 30 cycles and a final extension at 72°C for 10 min. Each PCR reaction yielded a single band detected in a 1.0% (w/v) agarose gel stained with ethidium-bromide [24]. PCR products were directly sequenced on an ABI 3370 DNA sequencer at Sangon Company (Shanghai, China) using a primer walking strategy. The complete mtDNA sequence of *P*. *cervi* was assembled using DNAStar software as a sequence editor [32].

**Table 1:** Primers for amplification of mt DNA genome of *P*. *cervi*.

Primer name (positions)	Sequence of primer (5′→3′)
PC1-F_nad5 (12177–12198)	TTDCKTCTCGNTTBGGKGATGT
PC1-R_cytb (1497–1519)	ARAAARTAYCACTCNGGCTTWAT
PC2-F_cytb (1110–1132)	TATTGRGCTGCTACDGTTTTGAC
PC2-R_nad2 (4623–4648)	CATCATATGACACCAACAATAATACC
PC3-F_nad2 (4054–4076)	TTTKTTTATGAGRTYTTTGTBGC
PC3-R_nad1 (5839–5963)	AYTCDCTYTCVGMCTCMSCRTAATC
PC4-F_nad1 (5350–5372)	CGTAAGGGKCCDAAHAAGGTTGG
PC4-R_cox1 (7603–7628)	CCAAARAAYCAAAAYAWATGYTGAAA
PC5-F_cox1 (7285–7307)	GTTGGKTGRACTTTTTATCCWCC
PC5-R_s-rRNA (9455–9474)	AGATAAGAACCGACCTGGCT
PC6-F_s-rRNA (9296–9318)	TTACCTYGGGGATAACTRRGTAA
PC6-R_nad6 (10851–10876)	GCACCACAHAAYTCMSTACARTAHCC
PC7-F_cox2 (10265–10292)	TAGCTCTGATAAGTCGTAACATGGTAAT
PC7-R_nad5 (12326–12343)	HGGAGCVCGCATHGCYTC

**Notes:** D = A/T/G; H = A/C/T; K = G/T; M = A/C; N = A/C/G/T; R = A/G; V = A/C/G; W = A/T; Y = T/C. The positions of primers in the study are based on the mt genome sequence of *P*. *cervi.*

### Prediction of protein-coding genes, tRNAs and genes for *rrn*L and *rrn*S

The ORF finder tool at NCBI (http://www.ncbi.nlm.nih.gov/gorf/gorf.html) was used to find protein-coding gene sequences, which were subsequently used to search for homologous digenean sequences deposited in the GenBank™ by using tBLASTn. The rhabditophoran platyhelminth genetic code [33] was specified. Gene boundaries were confirmed based on comparison and alignment with other published mt genomes of species in Fasciolidae, Opisthorchiidae and Paragonimidae [27], [28], [30], [31].

Putative tRNA genes were identified using the program tRNAscan-SE [34] and the online tool ARWEN [35] combined with observations and alignments by eye. Genes for large (*rrn*L) and small (*rrn*S) subunit ribosomal RNA genes were identified by comparison with the mt rRNA genes of *F*. *hepatica*, *C*. *sinensis*, *O*. *felineus*, *P*. *westermani* and other flatworms [27], [28], [31].

#### Phylogenetic analyses

DNA sequences of the 12 protein-coding genes were concatenated and imported into BioEdit [36]. After translation (using Translation Table 9 http://www.ncbi.nlm.nih.gov/Taxonomy/taxonomyhome.html/index.cgi?chapter=tgencodes#SG9), the concatenated amino acid sequences were aligned using Clustal [37], and then back-translated into nucleotide sequences to improve alignment. Phylogenetic trees were constructed using Bayesian analyses in MrBayes v3.1 [39] of concatenated sequences (nucleotides and inferred amino acid sequences) of the protein-coding genes in the mt genomes of *P*. *cervi* and 11 other digenean species (Table 2). The mt genome sequence of the cestode *Echinococcus granulosus* (NC_008075) was used as an outgroup.

**Table 2:** The arrangement and length (in bp) of protein-encoding genes and rRNA genes of *P*. *cervi* and other digenean species available in GenBank™.

Species	Accession number	Length (bp)	Order and Length (bp) of protein-coding genes and rRNA genes													
***Paramphist omum cervi***	KF47******5773	14,014	cox3 645	cytb******1113	nad4L 264	nad4 1281	atp6 516	nad2******873	nad1******897	nad3 357	cox1 1533	rrnL 992	rrnS 754	cox2******579	nad6 453	nad5******1581
***Fasciola hepatica***	NC_00******2546	14,462	cox3 642	cytb******1113	nad4L 273	nad4 1272	atp6 519	nad2******867	nad1******897	nad3 357	cox1 1533	rrnL 987	rrnS 766	cox2******603	nad6 453	nad5******1569
***Paragonimus westermani***	AF21******9379	14,965^1^	cox3 645	cytb******1119	nad4L 258	nad4 1263	atp6 513	nad2******867	nad1******903	nad3 357	cox1 1536	rrnL 987	rrnS 744	cox2******600	nad6 453	nad5******1584
***Opisthorchis felineus***	NC_01******1127	14,277	cox3 645	cytb******1116	nad4L 264	nad4 1278	atp6 516	nad2******870	nad1******903	nad3 357	cox1 1563	rrnL 994	rrnS 779	cox2******639	nad6 462	nad5******1605
***Clonorchis sinensis***	NC_01******2147	13,875	cox3******642	cytb******1113	nad4L 264	nad4 1278	atp6 516	nad2******873	nad1******903	nad3 357	cox1 1560	rrnL 998	rrnS 779	cox2******636	nad6 462	nad5******1605
***Trichobilharzia regenti***	NC_00******9680	14,838	cox3******651	cytb******1107	nad4L 261	nad4 1254	atp6******516	nad2******825	nad1******876	nad3 363	cox1 1536	rrnL 1016	rrnS 772	cox2******585	nad6******459	nad5******1599
***Schistosoma mekongi***	NC_00******2529	14,072^1^	cox3******654	cytb******1119	nad4L 264	nad4 1272	atp6******709	nad2******852	nad1******888	nad3 363	cox1 1533	rrnL 1019	rrnS 709	cox2******637	nad6******462	nad5******1593
***S. japonicum***	NC_00******2544	14,085^1^	cox3******645	cytb******1116	nad4L 264	nad4 1275	atp6******519	nad2******855	nad1******891	nad3 360	cox1 1527	rrnL 1004	rrnS 744	cox2******600	nad6******459	nad5******1587
***S. turkestanicum***	HQ28******3100	14,755	cox3******561	cytb******1110	nad4L 264	nad4 1263	atp6******513	nad2******837	nad1******918	nad3 363	rrnL 1047	cox1******1629	rrnS 761	cox2******585	nad6******450	nad5******1569
***S. haematobium***	NC_00******8074	15,003	cox3******666	cytb******1104	nad4L 261	nad4 1266	nad3******369	nad1******882	cox1******1542	rrnL 1055	rrnS 762	cox2******597	nad6******474	atp6******524	nad2******840	nad5******1584
***S. mansoni***	NC_00******2545	14,415^1^	cox3******654	cytb******1095	nad4L 261	nad4 1260	nad3******363	nad1******879	cox1******1533	rrnL 1055	rrnS 752	cox2******594	nad6******450	atp6******752	nad2******840	nad5******1584
***S. spindale***	NC_00******8067	16,901	cox3******666	cytb******1095	nad4L 255	nad4 1263	nad3******369	nad1******876	cox1******1548	rrnL 1056	rrnS 760	cox2******603	nad6******468	atp6******760	nad2******840	nad5******1587

1 Note that lengths given in GenBank for these entries do not include an undetermined portion of the long non-coding region.

In every case two runs, each of four chains, were specified. For the nucleotide alignment, the GTR+I+G model was as described previously [24], [38], partitioned by codon position. Bayesian analysis was run for 5,000,000 generations and sampled every 1000 generations. The first 25% of trees were omitted as burn-in and the remaining trees were used to calculate Bayesian posterior probabilities [39].

For the amino-acid alignment, MrBayes was allowed to determine the most appropriate model (“prset aamodelpr = mixed”), 2,000,000 generations were run and trees sampled every 500. The first 25% of trees were omitted as burnin.

## Results and Discussion

### 

#### General features of the mt genome of *P*. *cervi*

Lengths cited for some “complete” digenean mt genomes in GenBank are incorrect. They are instead the lengths of the coding portions. For these species, amplification and sequencing of non-coding regions proved impossible because of the presence of numerous repeats and other features. Complete mt genome lengths for these were inferred from Southern blotting experiments [27] or restriction fragment analysis. Thus the total length for the digenean *P*. *westermani* (AF219379) is around 21 kb [40] (14,965 bp in GenBank), and for *Schistosoma japonicum* (AF215860) and *S*. *mansoni* (AF216698), it is 16.5–24 kb [28], [41], [42] (around 14.5 kb stated in GenBank). However, other digeneans do possess small mt genomes. That of *S*. *spindale* (NC_008067) has a total length of 16,901 bp and *Trichobilharzia regenti* (NC_009680), also a member of the Schistosomatidae, has a very short non-coding region and a total length of 14,838 bp [19], [21]. *S*. *turkestanicum* is 14,755 bp [17].

The complete mtDNA sequence of *P*. *cervi* (deposited in GenBank, accession number KF475773) is 14,014 bp in length, within the range of typical sizes for metazoan mt genomes (14–18 kb). The mt genome of *P*. *cervi* is larger than that of *C*. *sinensis* (13,875 bp), but smaller than those of other digenean species available in GenBank™ to date (Table 2). It contains 12 protein-coding genes (*cox*1-3, *nad*1-6, *nad*4L, *atp*6 and *cyt*b), 22 transfer RNA genes and 2 ribosomal RNA genes (*rrn*L and *rrn*S) (Tables 2 and 3). All genes are transcribed in the same direction, which is consistent with other digeneans. The arrangement of protein-encoding genes in *P*. *cerv*i is the same as that of the *F*. *hepatica* [27], [28], *O*. *felineus* [30], *P*. *westermani*, *S*. *turkestanicum* [17], *S*. *japonicum* and *S*. *mekongi* mt genomes, but different from that seen in *S*. *haematobium*, *S*. *mansoni* and *S*. *spindale* [19].

**Table 3:** Positions and lengths of genes and regions of *P*. *cervi* mt genome, and start and stop codons for the protein-coding genes as well as anticodons for the tRNA genes (starting from *cox*3).

Gene/Region	Position 5′–3′	Size (bp)	Codons		Anti-codons	Intergenic Nucleotides (bp)^a^
			Start	Stop		
*cox*3	1–645	645	ATG	TAG		0
tRNA-His	647–715	69			GTG	3
*cyt*b	720–1832	1113	ATG	TAG		4
SNR	1833–1890	58				0
*nad*4L	1891–2154	264	ATG	TAG		0
*nad*4	2115–3395	1281	GTG	TAG		−40
tRNA-Gln	3398–3462	65			TTG	2
tRNA-Phe	3489–3553	65			GAA	26
tRNA-Met	3553–3615	63			CAT	−1
*atp*6	3616–4131	516	ATG	TAG		0
*nad*2	4139–5011	870	GTG	TAG		7
tRNA-Val	5014–5077	64			TAC	2
tRNA-Ala	5085–5154	70			TGC	7
tRNA-Asp	5165–5229	65			GTC	10
*nad*1	5233–6129	897	ATG	TAG		3
tRNA-Asn	6142–6207	66			GTT	12
tRNA-Pro	6208–6270	63			TGG	0
tRNA-Ile	6272–6334	63			GAT	1
tRNA-Lys	6344–6409	66			CTT	9
*nad*3	6410–6766	357	ATG	TAG		0
tRNA-Ser^AGN^	6785–6843	59			GCT	18
tRNA-Trp	6853–6915	63			TCA	9
*cox*1	6916–8460	1545	GTG	TAG		0
tRNA-Thr	8470–8534	65			TGT	9
*rrn*L	8535–9526	992				0
tRNA-Cys^b^	9527–9586	60			GCA	6
*rrn*S	9587–10340	754				5
*cox*2	10341–10919	579	ATG	TAG		0
*nad*6	10920–11372	453	GTG	TAG		0
tRNA-Tyr	11389–11455	67			GTA	16
tRNA-Leu^CUN^	11470–11536	67			TAG	14
tRNA-Ser^UCN^	11538–11609	72			TGA	1
tRNA-Leu^UUR^	11646–11710	65			TAA	36
tRNA-Arg	11713–11779	67			TCG	2
*nad*5	11780–13360	1581	GTG	TAG		0
tRNA-Gly	13365–13433	69			TCC	4
tRNA-Glu	13451–13515	65			TTC	17
LNR	13516–14014	499				0

**Notes**: ^a^ indicates length of intergenic gap (positive value) or overlap (negative value) between two adjacent genes. ^b^ the structure of tRNA-Cys may be three-armed with a DHU-replacement loop (9527–9586, 60 bp) or cloverleaf form (positions 9521–9588, 68 bp).

A 40 bp overlap between the 3′ end of *nad*4L and the 5′ end of *nad*4 was noted in *P*. *cervi*, similar to that of other digeneans.

### Protein-encoding genes

In total, 3,364 amino acids are encoded by the *P*. *cervi* mt genome. The nucleotide composition in *P. cervi* was biased toward G and T, which is similar to that of the digeneans *F*. *hepatica*, *O*. *felineus*, *C*. *sinensis*, *P*. *westermani* and the outgroup cestode, *E*. *granulosus*, but is slightly different from *S*. *turkestanicum*, *S*. *japonicum* and other schistosomes, which are biased toward A and T. In the protein-coding genes of *P*. *cervi*, strong bias against the usage of C (8.76%, on average) and strong bias in favor of the usage of T (47.77%, on average) were observed. The frequency of usage for G (27.46%, on average) was higher than that for A (16.01%, on average) (Table 4).

**Table 4:** Comparisons of A+T content of protein-coding genes and rRNA genes of mt genome of *P*. *cervi*.

Gene	A (%)	G (%)	T (%)	C (%)	A+T (%)
*cox*3	15.04	27.75	50.08	7.13	65.12
*cyt*b	16.98	28.03	45.82	9.16	62.80
*nad*4L	17.42	29.17	46.97	6.44	64.39
*nad*4	15.53	26.39	48.71	9.37	64.25
*atp*6	16.28	24.22	49.81	9.69	66.09
*nad*2	14.89	25.66	51.89	7.56	66.78
*nad*1	16.24	28.93	47.21	7.61	63.45
*nad*3	15.13	27.73	50.14	7.00	65.27
*cox*1	15.79	27.20	45.79	11.22	61.58
*rrn*L	25.96	26.77	37.63	9.63	63.59
*rrn*S	23.90	27.77	36.85	11.48	60.75
*cox*2	19.34	28.67	41.80	10.19	61.14
*nad*6	14.79	28.92	49.23	7.06	64.02
*nad*5	16.51	27.83	47.56	8.10	64.07
LNR	25.70	27.51	38.55	8.23	64.26
SNR	20.69	31.03	41.38	6.90	62.07

The most common inferred start codon for mt protein-encoding genes of digenean species is ATG, followed by GTG (e.g. *F*. *hepatica* [27], *O*. *felineus* and *C*. *sinensis* [30], *S*. *turkestanicum* [17], *S*. *spindale* and *S*. *haematobium* [19]). GTG was also a frequent initiation codon (5/12) for the mt protein-encoding genes of *P*. *cervi*. It is interesting that the stop codon TAG was used for all the mt protein-coding genes of *P*. *cervi*. This is unusual, because another termination codon, TAA, is often observed in other digeneans.

### Transfer RNA (tRNA) genes

**Figure 1:**
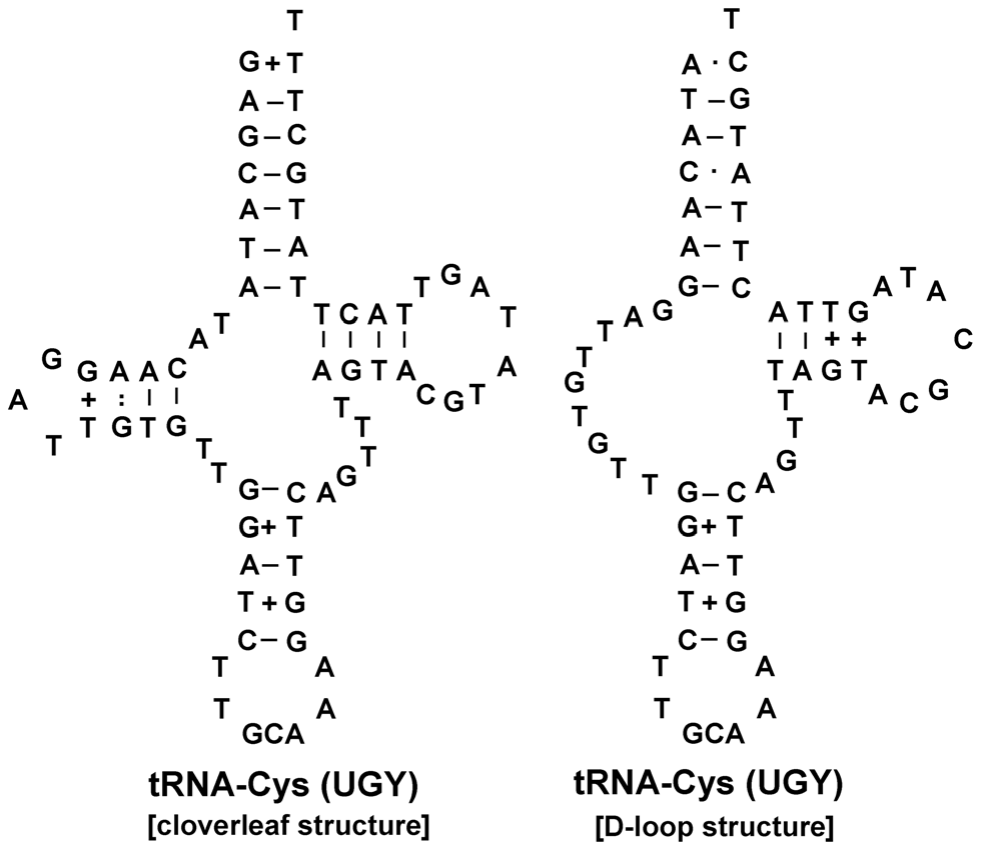
Two possible structures of tRNA-Cys (cloverleaf structure or D-loop).

Except for tRNA-Ser1^(AGN)^ and tRNA-Cys, all tRNA genes appear to exhibit the standard cloverleaf structure. The predicted secondary structure of the serine tRNA^(AGN)^ contains the TΨC arm but lacks the DHU arm (terminology follows Wolstenholme, 1992) [43], a situation which is also found in *O*. *felineus* [30] and some other digeneans. For the cysteine tRNA, a four-armed structure is feasible, but so is a three-armed structure with a DHU-replacement loop (Fig. 1). It is noteworthy that tRNA-Glu and tRNA-Gly have switched positions in *P*. *cervi* relative to the situation in *F*. *hepatica*, *P*. *westermani* and the opisthorchiids (*O*. *felineus*, *O*. *viverrini* and *C*. *sinensis*), suggesting that this change in tRNA gene position could provide an important phylogenetic signal [19].

### Ribosomal RNA genes

The *rrn*L (16S ribosomal RNA) and *rrn*S (12S ribosomal RNA) genes of *P. cervi* were identified by sequence comparison with those of *F*. *hepatica*, *O*. *felineus*, and *Schistosoma* spp. These two genes are separated by tRNA-Cys. The sizes of the *rrn*L and *rrn*S genes were 986 and 749 bp, respectively, and their A+T content was 63.59% and 60.75%, respectively, which are the lowest among the digeneans studied to date (Tables 2 and 4).

### Non-coding regions

Non-coding regions exist in the mt genomes of many parasitic flatworms, but the locations of these relative to major genes tend to be rather variable. It is usual to recognize two such non-coding regions in digeneans: long and short non-coding regions (LNR and SNR) that are often separated by one or more tRNA genes. A common feature of LNRs is the presence of long repeats. Such features are found in *F*. *hepatica*, most or all *Schistosoma* species (but await further characterization in several species) and in *P*. *westermani* (for which the LNR also awaits full characterization). In other species, notably in the genera *Opisthorchis* and *Clonorchis*, the non-coding regions lack strong structures, such as large repeats. There does not seem to be a strong phylogenetic element to length and structure of the LNR. In *P*. *cervi*, there is a short non-coding region (SNR) (58 nucleotides), lacking any notable features and located between *cyt*b and *nad*4L. A long non-coding region (LNR) (499 nucleotides), is observed between tRNA-Glu and *cox*3 (Tables 3 and 4). Short homopolymer tracts (< 8 nt) and short microsatellite-like tracts – e.g. (AT)_n_ – are present in this region, but there are no long direct or inverted repeats, nor any similarities with the SNR (Tables 3 and 4). Although the replication process(es) of mt DNA of digeneans is unclear, it is not difficult to predict that the AT-rich non-coding region might be involved in the initiation of replication [9], [17], [19], [44].

### Phylogenetic analyses

**Figure 2:**
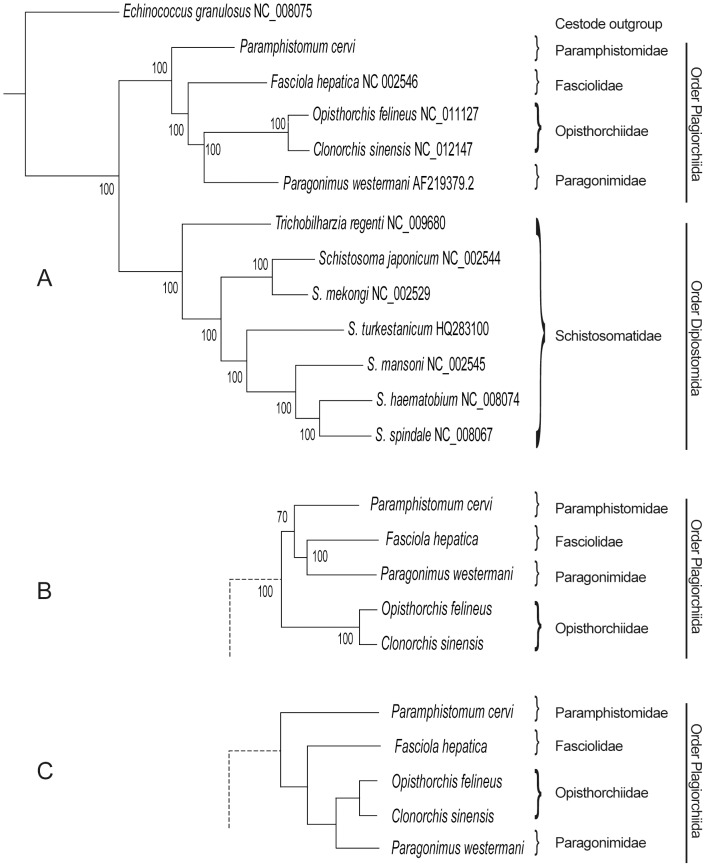
Inferred phylogenetic relationship among the digenean species. Trees were inferred using MrBayes v3.1. A, tree inferred from concatenated nucleotide sequences of 12 protein-coding genes, using the cestode *E*. *granulosus* as the outgroup. Posterior support values are given at nodes. See text for more details. B, tree inferred from concatenated amino acid sequences. Only the portion of the tree (members of the order Plagiorchiida) that differs from that in A is shown. C, tree of members of the Plagiorchiida according to phylogeny proposed by Olson et al (2003) [47].

Some systematic and population genetic studies have been completed based on genetic markers in the mt genomes of flukes [16], [17], [19], [21], [24], [26], [30], [45]. So far, the full-length mt genomes of 12 digenean species have been determined and characterized, and these have been used in the phylogenetic study. Using complete mt sequences for phylogenetic analyses is more reliable according to the study of Waeschenbach et al (2012), who confirmed that alignments of >10,000 nucleotides from mtDNAs can provide a rich resource for phylogeny construction, hypothesis-testing and interpretation of the evolution of the major lineages of tapeworms [46]. Now that we have a complete mt genome from a member of the Paramphistomidae, we can begin to explore this possibility for the digeneans. The tree inferred from concatenated nucleotide sequences of the 12 protein-coding genes is shown in Fig. 2A. All nodes are supported by very high posterior probabilities (100%). Two large clades are apparent: one contains seven members of the Family Schistosomatidae (Order Diplostomida – following [47]) and the other includes five members representing four families within the Order Plagiorchiida. Fig. 2B reveals the corresponding tree inferred from amino-acid sequences (only species within the Plagiorchiida are shown: the tree for the members of the Diplostomida was identical with that in Fig. 2A). MrBayes indicated that the most appropriate substitution model for the amino-acid alignment was “cprev”, originally developed for proteins encoded by chloroplast genomes [48]. For the Diplostomida, phylogenetic relationships depicted in Fig. 2A exactly match those previously reported (e.g. Morgan et al, 2003) [49]. For members of the Plagiorchiida, the situation is a little more complicated. Fig. 2C depicts relationships among the four plagiorchiid families abstracted from the phylogeny in Olson et al (2003) [47]. According to this, the sequence of families in order of increasingly derived status is: Paramphistomidae, Fasciolidae, Opisthorchiidae and Paragonimidae. This arrangement was seen in the tree based on nucleotide sequences (Fig. 2A), but not in the tree inferred from amino-acid sequences (Fig. 2B), in which a clade containing Fasciolidae and Paragonimidae was strongly supported and *P*. *cervi* was sister to this clade (in 70% of trees). In 30% of trees, *P*. *cervi* was placed basal to the other plagiorchiids (as in Fig. 2A), but even in these trees, the clade containing *Fasciola* and *Paragonimus* was strongly supported. Speculation as to the cause of this is premature. Our sampling of the Order Plagiorchiida is very sparse and sequences from many additional digenean mt genomes will be needed before we can be sure that we have stable and defensible phylogenetic hypotheses.

## Summary

In conclusion, the present study determined the complete mt genome sequence of *P. cervi*, which possesses the same gene order (except for tRNA-Glu and tRNA-Gly) as most other digeneans, consisting of 12 protein-coding genes, 2 rRNA genes and 22 tRNA genes. Phylogenetic trees, based on sequences of protein-coding genes, could identify the two orders represented (Diplostomida and Plagiorchiida). For members of the Diplostomida, relationships are exactly as expected from other studies. For the five members of the Plagiorchiida, results are not as consistent, but our sampling of this clade is very sparse and additional sequences are needed. The complete mtDNA sequence of *P. cervi* will add the knowledge to digenean mitochondrial genomics. It will also provide an important resource for the studies of inter- and intra-specific variation of the Paramphistomidae and a resource for comparative mitochondrial genomics and systematic studies of digeneans.
